# Novel orthohepeviruses in wild rodents from São Paulo State, Brazil

**DOI:** 10.1016/j.virol.2018.03.025

**Published:** 2018-06

**Authors:** William Marciel de Souza, Marilia Farignoli Romeiro, Gilberto Sabino-Santos, Felipe Gonçalves Motta Maia, Marcilio Jorge Fumagalli, Sejal Modha, Marcio Roberto Teixeira Nunes, Pablo Ramiro Murcia, Luiz Tadeu Moraes Figueiredo

**Affiliations:** a Virology Research Center, Ribeirão Preto Medical School, University of São Paulo, Av. Bandeirantes, 3900, Monte Alegre, 14049-900 Ribeirão Preto, SP, Brazil; b MRC-University of Glasgow Centre for Virus Research, Glasgow, United Kingdom; c Laboratory Institute of Biomedical Sciences, University of São Paulo, São Paulo, Brazil; d Center for Technological Innovations, Evandro Chagas Institute, Ministry of Health, Ananindeua, Pará, Brazil

**Keywords:** Hepatitis E virus, Viral hepatitis, *Orthohepevirus*, *Hepeviridae*, Rodent-borne viruses

## Abstract

The *Hepeviridae* comprise single-stranded positive-sense RNA viruses classified into two genera, *Orthohepevirus* and *Piscihepevirus*. Orthohepeviruses have a wide host range that includes rodents, but previous studies had been restricted to rodents of the Muridae family. In this study, we applied a high-throughput sequencing approach to examine the presence of orthohepeviruses in rodents from São Paulo State, Brazil. We also used RT-PCR to determine the frequency of orthohepeviruses in our sampled population. We identified novel orthohepeviruses in blood samples derived from *Necromys lasiurus* (1.19%) and *Calomys tener* (3.66%). Therefore, our results expand the host range and viral diversity of the *Hepeviridae* family.

## Highlights

• Genomic and phylogenetic characterization of new orthohepevirus.
• Expansion of the viral diversity and host orthohepevirus.
• First report of orthohepevirus in *Cricetidae* rodents.

## 1 Introduction

Hepatitis E virus (HEV) is a single-stranded, positive-sense RNA virus with a genome of 6.6–7.3 kb in length that belongs to the genus *Orthohepevirus* within the *Hepeviridae* family (Purdy et al., 2017, Smith et al., 2014). HEV is a leading cause of acute sporadic hepatitis and fulminant hepatic failure in humans (Kamar et al., 2014). Human infections can be acquired via contaminated water, consumption of undercooked or raw meat, direct contact with infected animals, and environmental contamination by animal manure run-off (Doceul et al., 2016). The disease caused by HEV is generally self-limiting with mortality rates of 0.5–3% in adults. In addition, recent studies have shown that HEV can infect neural cell lines, suggesting that HEV can cause in rare cases extrahepatic manifestations (Kamar et al., 2014, Zhou et al., 2017).

HEV is prevalent in a wide range of mammals and has been reported in developing and industrialized countries (Kamar et al., 2014). Our knowledge about the number of hosts harboring infections by members of the *Hepeviridae* family have been dramatically expanded in recent years, as well as the diversity of virus variants (Doceul et al., 2016). The *Hepeviridae* family is divided into two genera, the *Piscihepevirus* genus that includes a single species detected in salmonids (Smith et al., 2014), and the *Orthohepevirus* genus, comprised of four species: *Orthohepevirus A* includes viral strains known to infect humans, swine, camels, sheep, boars, deer, rabbits, and mongooses as well as rats; *Orthohepevirus B* comprises viral strains reported in birds; *Orthohepevirus C* includes viral strains described in rats, shrews, bandicoots, mink and ferrets, and *Orthohepevirus D* contains viral strains identified in bats (Purdy et al., 2017, Smith et al., 2014, Spahr et al., 2017). Currently, HEV exhibits a broad range of hosts including rodents of the Muridae family (Doceul et al., 2016, Lack et al., 2012, Li et al., 2013, Purcell et al., 2011). Here, we used a high-throughput sequencing (HTS) approach to identify and characterize orthohepeviruses present in blood samples derived from wild rodents in the northeastern region of São Paulo State, Brazil.

## 2 Materials and methods

### 2.1 Rodent samples

Between 2008 and 2013, blood samples were collected from 647 wild rodents in the rural area of Ribeirão Preto, São Paulo State, Brazil. Based on morphological features and cytochrome-b gene, the rodents were classified in five different species, *Akodon montensis*, *Calomys tener*, *Oligoryzomys nigripes*, *Necromys lasiurus* and *Mus musculus* (Bonvicino et al., 2008, Salazar-Bravo et al., 2013). Then, samples were divided into pools based on species and collection date (Table 1).

**Table 1:** Information of sample pools used in this study.

**Pool**	**Species**	**N**^a^	**Collection date**	**Number of reads**
1B	*Akodon montensis*	55	2008	27,569,342
2B	*Akodon montensis*	55	2008	23,439,326
3B	*Akodon montensis*	41	2009	16,698,848
4B	*Akodon montensis*	48	2012–2013	18,783,944
5B	*Calomys tener*	38	2008	27,017,352
6B	*Calomys tener*	37	2008	23,972,304
7B	*Calomys tener*	34	2009, 2012–2013	15,679,756
8B	*Necromys lasiurus*	59	2008	22,989,548
9B	*Necromys lasiurus*	59	2008	9252,300
10B	*Necromys lasiurus*	58	2008	18,213,066
11B	*Necromys lasiurus*	52	2009	22,210,888
12B	*Necromys lasiurus*	24	2012–2013	25,122,228
13B	*Oligoryzomys nigripes*	43	2008–2009	15,813,430
14B	*Oligoryzomys nigripes*	20	2012–2013	24,796,054
15B	*Mus musculus*	24	2008–2009	16,661,928

a N: number of individual samples per pool.

### 2.2 Preparation of pools, viral genome sequencing and assembly

Viral RNA of pooled samples were extracted using the QIAamp viral RNA mini kit (Qiagen, USA) and stored at −80 °C. Subsequently, nucleic acids were quantified using a Qubit® 2.0 Fluorometer (Invitrogen, Carlsbad, USA) and the purity and integrity of nucleic acid of samples were measured using an Agilent 2100 Bioanalyzer (Agilent Technologies, USA). cDNA synthesis was performed using SuperScript II (Invitrogen, Carlsbad, USA). Sequencing was performed using the TruSeq RNA sample preparation kit in an Illumina HiSeq. 2500 instrument (Illumina, USA) with a paired-end and 150-base-read protocol in *RAPID* module. Sequencing reads were assembled *de novo* using the metaViC pipeline as previously described (de Souza et al., 2017).

### 2.3 Genome characterization and phylogenetic analysis

Viral genomes were assessed for genome size and ORF prediction with Geneious 9.1.2 (Biomatters, New Zealand). ORFs were confirmed using the BLASTX database and based on position and nucleotide sequence similarities on NCBI database. Also, the presence of protein domains was screened using Motif Scan (http://myhits.isb-sib.ch). The nucleotide sequences determined in this study were deposited in GenBank under the accession numbers MG021328 to MG021330.

A maximum likelihood (ML) phylogenetic tree was inferred using a protein alignment of 423 amino acids of the RNA-dependent RNA polymerase region – RdRP (ORF1-1249 to ORF1-1671, numbered relative to the sequence of the HEV Burma isolate, GenBank accession M73218). In addition, we added partial sequences of RdRp region selected in final alignment, including sequences with 86 aa from Mink (GenBank No. KC802090), 108 aa from bats (GenBank No. JQ001744-6 and 8), 120 aa from Fox (GenBank No. KC692370), and 202 aa from Calomys HEV, which was identified in this study. The short sequences did not adversely affect the phylogeny analysis, and we observed the same topology in phylogenetic using the complete sequences of RdRp (Figure Supplementary 1), as showed in previous study (Smith et al., 2014). Therefore, the final alignment contained sequences of the viruses identified in the present study together with sequences obtained from representative members of the *Hepeviridae* family (Smith et al., 2014). The multiple sequence alignment (MSA) was carried out using Muscle v3.8 (Edgar, 2004) and the ML tree was inferred using IQ-TREE version 1.6.0 software using LG+I+G4 substitution model with 1000 ultrafast bootstraps. The LG+I+G4 model was the best-fit model out of 144 reversible amino acids substitution models based on Bayesian Information Criterion (Kalyaanamoorthy et al., 2017, Nguyen et al., 2015). Statistical support for individual nodes of the phylogenetic tree was estimated using the bootstrap value. The phylogenetic tree was visualized using the FigTree software v.1.4.2. Also, the amino acid evolutionary distances among clades were estimated with RdRp using the p-distance values. Thus, the number of amino acid differences per site from averaging over all sequence pairs between groups were calculated based on 56 sequences with 423 amino acid in length. All ambiguous positions were removed for each sequence pair. Standard error estimations were calculated with the bootstrapping method (1000 replicates) using the MEGA v.6 program (Tamura et al., 2013). The results of the amino acid distances among clades were presented in a box and whisker plot (Whiting et al., 2008).

### 2.4 Frequency of new orthohepeviruses

To determine the authenticity and frequency of viral genomes of novel orthohepeviruses, we designed primer sets to specifically amplify an ~800 bp long sequence between ORF 1 and ORF2 gene of the viruses identified in this study (forward primer: 5′-GAGGGCCGGGCCTC(T/C)TTTGTT-3′; reverse primer: 5′ -CAGTGGGTTTAACGAGGCGGCAT-3′). Then, the viral RNA of individual rodents samples was extracted using the QIAamp viral RNA extraction kit (Qiagen, Germany) and converted to cDNA using Superscript III (Invitrogen, USA) with random hexamers (Invitrogen, USA), following the manufacturer's instructions. Subsequently, PCR was performed using Platinum SuperFi DNA Polymerase (Thermo Fisher Scientific, USA), following the manufacturer's instructions. Cycling conditions were: 98 °C for 30 s followed by 35 cycles at 98 °C for 10 s, 70 °C for 10 s and 72 °C for 30 s, followed by a final extension of 72 °C for 5 min. Amplicons were visualized by gel electrophoresis in 1.5% agarose gels. All PCR products were verified by dideoxy sequencing using ABI 3730 genetic analyzer (Applied Biosystems, USA).

## 3 Results and discussion

**Fig. 1:**
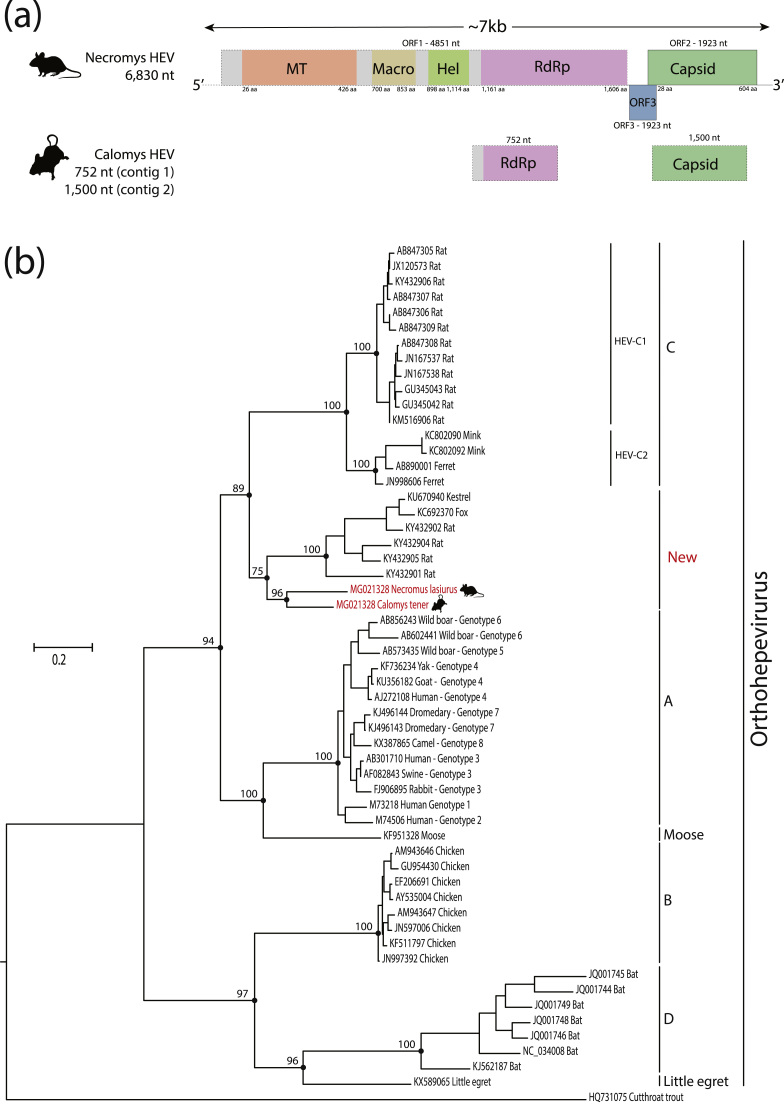
(a) Genome organization of the complete coding sequence of Necromys HEV and a partial genome of Calomys HEV. The length of the determined nucleotide sequences of the viral sequences are shown at the top. Solid-lined boxes and dashed-lined arrows indicate complete or partial sequence of ORFs, respectively. ORF1 encodes non-structural proteins including putative functional domains. MT: methytransferase; Macro: Macro domain profile; Hel: helicase; RdRp: RNA-dependent RNA polymerase. ORF2 (green) encodes capsid protein and ORF3 (blue) encodes a small phosphoprotein with a multi-functional C-terminal region. (b) Maximum likelihood phylogenetic tree showing the evolutionary relationships of viruses identified in our study with representatives of the *Hepeviridae* family. Phylogenies are midpoint rooted for clarity of presentation. The scale bar indicates evolutionary distance in numbers of substitutions per amino acid site. Bootstrap values of 1000 replicates are shown in principal nodes. HEV sequences generated in this study are shown in red. (For interpretation of the references to color in this figure legend, the reader is referred to the web version of this article.)

The HTS analysis of fifteen pools of blood rodents generated a total of 9252,300 to 27,569,342 paired-end reads (Table 1). After assembly, we identified a nearly complete genome of a novel strain of HEV in the blood pool (Pool 5B – Table 1) of Hairy-tailed bolo mouse (*Necromys lasiurus*) with 6830 nucleotides (nt) and two partial genomes of other strain, one from ORF1 gene with 752 nt and another from capsid gene of 1500 nt in the blood pool (Pool 12B – Table 1) of Delicate vesper mouse (*Calomys tener*), which were tentatively been designated as Necromys HEV (NeHEV) and Calomys HEV (CaHEV), respectively (Fig. 1**a**). The nearly complete NeHEV sequence was 6830 nucleotide long and exhibits a typical genome organization associated with members of the *Orthohepevirus* genus, which contains three open-reading frames (ORFs) that encode for the non-structural polyprotein (ORF1), Capsid (ORF2) and ORF 3 protein (Ding et al., 2017, Kamar et al., 2014, Purdy et al., 2017). Analysis of the non-structural polyprotein and capsid using BLASTX showed that the NeHEV and CaHEV shared between 67% and 76% amino acid identity with strains of orthohepevirus described in rodents from China (GenBank No. KY432901), and less than 62% amino acid identity with other representative orthohepeviruses.

**Fig. 2:**
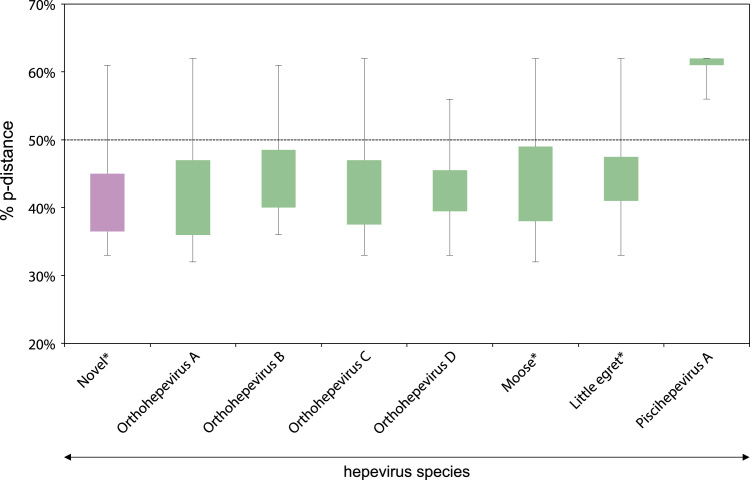
Amino acid p-distance of the novel clade (orthohepevirus from rodents derived from this study, kestrel, fox, and rodents from China) and representatives viruses of *Hepeviridae* family. This analysis was based on an alignment of the RdRp protein (ORF1–1249 to ORF1–1671, numbered relative to the sequence of the HEV Burma isolate, GenBank accession M73218). The ends of the box represent the upper and lower quartiles, as well as the box spans the interquartile range. The median is showed by a white vertical line inside the box, and the whiskers are the two lines outside the box that extend to the highest and lowest p-distance. The percentages of p-distance are shown in Y-axis, and the representative members of *Hepeviridae* family are shown in X-axis. The dashed-line showed the 50% of p-distance. The asterisks indicate the unrecognized species by ICTV. The novel clade is highlighted in pink color. (For interpretation of the references to color in this figure legend, the reader is referred to the web version of this article.)

Phylogenetic analysis using the RdRP gene showed that NeHEV and CaHEV did not cluster in any of the four species previously recognized for genus *Orthohepeviridae*. Instead, they formed a unique and monophyletic clade with orthohepeviruses derived various animal species including a fox captured in the Netherlands (Bodewes et al., 2013), a kestrel from Hungary (Reuter et al., 2016) and other strains of orthohepeviruses identified in rodents from China (Fig. 1**b**). This novel clade is more closely related to the *Orthohepevirus C* species that include viruses derived from Rodentia, Carnivora, and Soricomorpha (Smith et al., 2014). However, the novel clade that included the Necromys HEV and Calomys HEV have p-distances of less than 0.5 to other species of genus *Orthohepevirus* as showed in Fig. 2 (Smith et al., 2014). Therefore, based on our analysis and the species demarcation criteria of the *Hepeviridae* family (Purdy et al., 2017, Smith et al., 2014), we propose that the NeHEV and CaHEV should constitute strains of a new orthohepevirus species within the genus *Orthohepevirus*.

To determine the prevalence of NeHEV and CaHEV in *Necromys lasiurus* and *Calomys tener*, individual samples were screened by RT-PCR. A total of 3 out of 252 samples (1.19%) derived from *Necromys lasiurus* and 4 out of 109 (3.66%) samples derived from *Calomys tener* were PCR positive. As expected, positive samples were identified only in pools where the viruses had been identified by HTS.

The orthohepeviruses closely related to NeHEV and CaHEV were recently identified in common kestrels (*Falco tinnunculus)* and red-footed falcons (*Falco vespertinus*) from Hungary (Reuter et al., 2016). Furthermore, a previous study reported the mammalian HEV in fecal samples of Himalayan griffons (*Gyps himalayensis*) of Beijing Zoo in China (Li et al., 2015). Based on the high orthohepevirus viral load observed in kestrel feces it was suggested that the gastrointestinal tract might be the site of viral replication (Reuter et al., 2016). However, a dietary source of virus should also be considered as those birds feed on small mammals including shrews and rodents. In this novel clade, the orthohepevirus identified in fecal samples of fox (*Vulpes vulpes*) in Netherlands, suggests that this virus could be derived from their prey (e.g., rats) (Bodewes et al., 2013). Our results showed that rodents presented viremia, suggesting that viral replication might take place in these hosts and further indicate that rodents might act as natural reservoirs for this virus. Furthermore, infection of carnivore birds and foxes through the dietary route and presence of viruses in the feces could play an important role in the spread of this virus in the environment, but this feature needs to be elucidated.

In the current study, we detected the novel HEV in 1.19% samples of Hairy-tailed bolo mice and 3.66% in samples of Delicate vesper mice from a rural area in São Paulo State, Brazil. Comparative genomic and phylogenetic analyses showed that NeHEV and CaHEV represent previously unrecognized orthohepevirus species. Our results expanded the ecology of HEV among wild rodents and now include members of the *Cricetidae* family.

## Appendix A Supplementary material

Supplementary material **Supplementary Figure 1.** Maximum likelihood phylogenetic tree showing the evolutionary relationships of virus identified in our study with representatives of the *Hepeviridae* family using only complete RdRp protein. Phylogenies are midpoint rooted for clarity of presentation. The scale bar indicates evolutionary distance in numbers of substitutions per amino acid site. Bootstrap values of 1000 replicates are shown in principal nodes. HEV sequence generated in this study is shown in red.
