# Supplementary figures and images for: Novel orthohepeviruses in wild rodents from São Paulo State, Brazil
Source: Virology. 2018 Jun;519:12–6. doi: 10.1016/j.virol.2018.03.025 (PMC5998382; doi:10.1016/j.virol.2018.03.025)

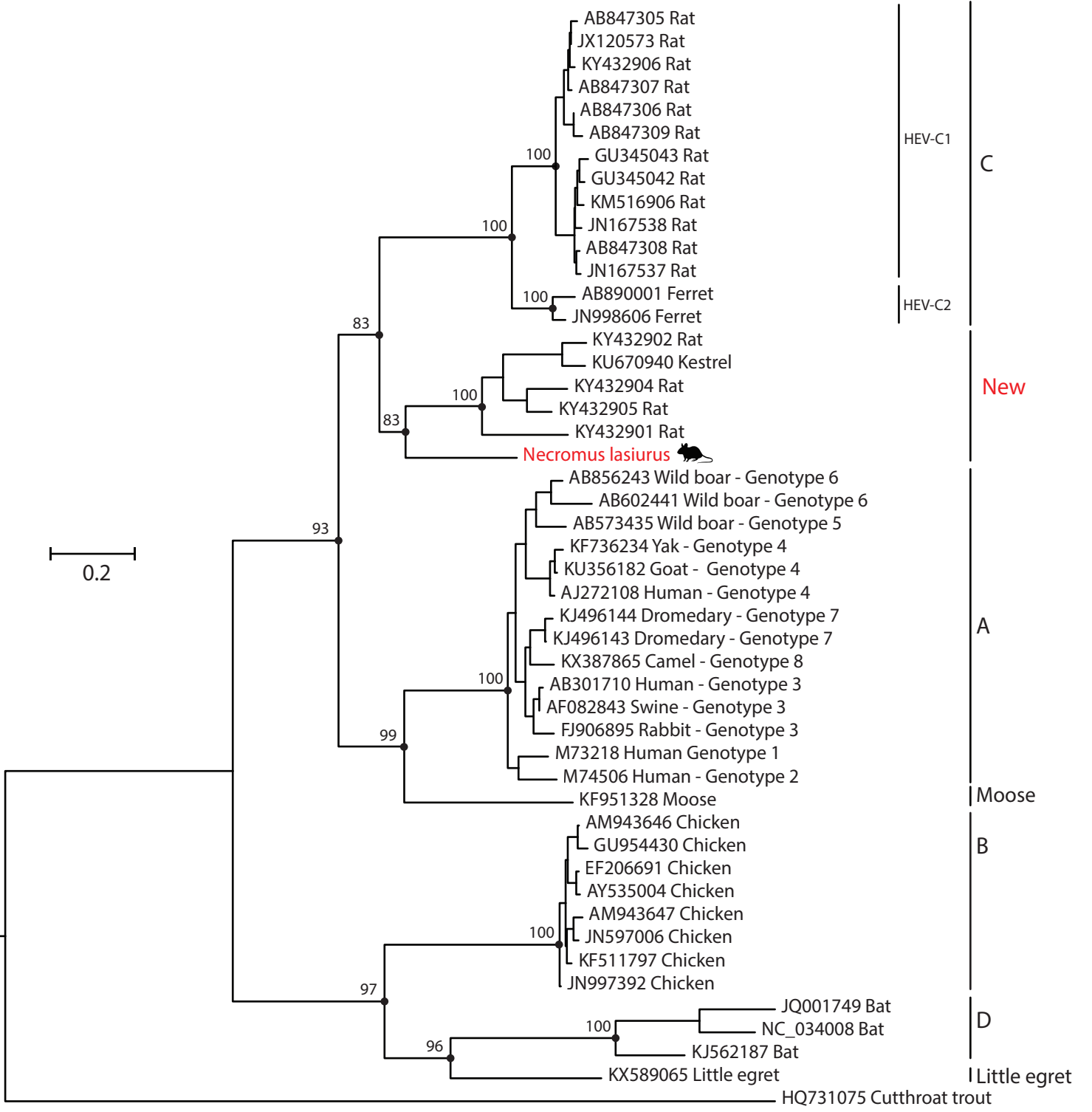

Orthohepevirus

Supplement: Supplementary file 1 — Supplementary material Supplementary Figure 1. Maximum likelihood phylogenetic tree showing the evolutionary relationships of virus identified in our study with representatives of the Hepeviridae family using only complete RdRp protein. Phylogenies are midpoint rooted for clarity of presentation. The scale bar indicates evolutionary distance in numbers of substitutions per amino acid site. Bootstrap values of 1000 replicates are shown in principal nodes. HEV sequence generated in this study is shown in red. [file mmc1.pdf]
